# Factors Associated with Self-Reported HBV Vaccination among HIV-Negative MSM Participating in an Online Sexual Health Survey: A Cross-Sectional Study

**DOI:** 10.1371/journal.pone.0030609

**Published:** 2012-02-17

**Authors:** Jonathan E. Matthews, Rob Stephenson, Patrick S. Sullivan

**Affiliations:** 1 Department of Epidemiology, Emory University Rollins School of Public Health, Atlanta, Georgia, United States of America; 2 Hubert Department of Global Health, Emory University Rollins School of Public Health, Atlanta, Georgia, United States of America; Asociacion Civil Impacta Salud y Educacion, Peru

## Abstract

**Background:**

A substantial proportion of men who have sex with men (MSM) in the United States remain unvaccinated against hepatitis B. We sought to understand which factors are associated with vaccination among HIV-negative MSM.

**Methodology/Principal Findings:**

Data were from a 2010 web-based survey of adult MSM. We calculated the prevalence of self-reported hepatitis B vaccination among 1,052 HIV-negative or HIV-untested men who knew their hepatitis B vaccination status, and used multivariate logistic regression to determine associated factors. 679 (64.5%) MSM reported being vaccinated. Younger men were more likely to report being vaccinated than older men, and there was a significant interaction between age and history of hepatitis B testing. Men with at least some college education were at least 2.1 times as likely to be vaccinated as men with a high school education or less (95% CI = 1.4–3.1). Provider recommendation for vaccination (aOR = 4.2, 95% CI = 2.4–7.4) was also significantly associated with receipt of vaccination.

**Conclusions/Significance:**

Providers should assess sexual histories of male patients and offer those patients with male sex partners testing for hepatitis infection and vaccinate susceptible patients. There may be particular opportunities for screening and vaccination among older and more socioeconomically disadvantaged MSM.

## Introduction

As part of their sexually transmitted disease treatment guidelines, the US Centers for Disease Control and Prevention recommends that all MSM with multiple sex partners should be vaccinated against hepatitis B [1], [2]. A hepatitis B vaccine has been available in the United States since licensure in 1981, and vaccination has been recommended for MSM, as well as other high-risk adults, healthcare workers, and infants born to hepatitis B-positive mothers since 1982 [3]. Vaccine recommendations were expanded in 1991 to cover all infants, as well as adolescents who engage in high-risk sexual and non-sexual behaviors [4], and, by 1997, all children under the age of 18 [5]. Hepatitis B vaccine is not administered to individuals who have serological evidence of previous infection.

Although heterosexual contact remains the primary mode of transmission of hepatitis B among adults, the disease burden associated with male-male sexual contact is substantial: as of 2005, one in four incident cases of hepatitis B was reported among MSM [1]. The prevalence of hepatitis B infection among MSM has been significantly reduced in part due to the development of a hepatitis B vaccine: in the 1970s, 3 in 5 MSM had serologic evidence of prior infection [6], [7]; after 1981, when the vaccine was licensed, less than one in three MSM has ever been infected with hepatitis B [8]–[14].

Despite substantial reductions in the prevalence of infection, nationally representative surveys have indicated that fewer than one in five young MSM has serologic evidence of vaccination against hepatitis B [11], [12]. By contrast, self-reported hepatitis B vaccination rates are often much higher: Finlayson et al reported in 2011 that 48% of MSM interviewed for the National HIV Behavioral Surveillance System reported ever having been vaccinated against hepatitis B [15]. Self-reported vaccination rates among MSM in select cities across the United States have been more variable, ranging from 3% to 70% [13], [14], [16]–[18].

A number of recent studies have presented analyses that describe demographic and behavioral factors associated with receipt of hepatitis B vaccination among MSM. However, many of these studies are from limited geographic areas, from restricted age groups, or are confined to respondents from urban areas. Thus, the results of past studies may not be reflective of patterns of self-reported vaccination among MSM across the United States. Multisite studies of self-reported hepatitis B vaccination prevalence and factors associated with vaccination have been conducted among younger MSM in larger US cities [11], [12]. In addition, a number of previous studies have not evaluated the statistical significance of covariates in the context of a multivariate model [8], [13], [17].

To address these gaps in previous studies, we used data from a national online survey of MSM to describe the prevalence of hepatitis B vaccination in HIV-negative MSM. Using logistic regression models, we evaluated factors associated with receipt of self-reported vaccination among these men. In addition, we described self-reported reasons for lack of vaccination among MSM who were not vaccinated against hepatitis B.

## Methods

### Ethics Statement

The present study received institutional review board exemption from the Emory University Institutional Review Board (IRB00044470). No identifying information was collected from respondents. Prior to being shown any study-related questions, all participants were informed they could skip any question they did not feel comfortable answering, and most questions also allowed participants to indicate that they did not know the answer or preferred not to answer.

We recruited men to a web-based sexual health survey through banner ad recruitment. We displayed banner advertisements for a men's health survey to men living in the United States who reported being interested in men on the social networking Internet site Facebook (http://www.facebook.com), and to members of the site Black Gay Chat (http://www.bgclive.com) from October 28 to December 12, 2010. Men who clicked through the banner advertisement were forwarded to a web page with a series of screening questions. Individuals were eligible for participation in the survey if they were male and 18 years of age or older. All female respondents, as well as those male respondents who were under 18 years of age or who did not specify their age, were screened out of our study and were not presented with any additional study-related questions. Consenting study participants were asked a series of questions related to demographic characteristics (race and ethnicity; educational, employment and insurance status; state of residence); experiences as a gay or bisexual man; lifetime history of sexual partners; characteristics of the participant's most recent male sex partner (MRMSP) [including demographic characteristics and information on the partner's HIV status]; sexual (oral and anal intercourse; condom use) and non-sexual (drug use) risk behaviors engaged in at last sex with the MRMSP; HIV knowledge, testing behaviors and status; having had a care visit with a healthcare provider in the last 12 months; and hepatitis B diagnosis, testing and vaccination status.

Our primary analytic objective was to determine the prevalence of and factors associated with self-reported receipt of hepatitis B vaccination among MSM. Therefore, participants were excluded from our analysis if they reported having sex with only women, reported never having sex, or did not specify a history of sexual partners. In particular, we were interested in evaluating the extent of hepatitis B vaccination among HIV-negative MSM. Therefore, MSM who were HIV-positive or who did not specify or know whether they had ever been tested for HIV, as well as men who had been tested for HIV but chose not to report their HIV status, were excluded from our analysis.

Participants who remained in our analysis sample were included in univariate analysis of all independent predictors. To assess hepatitis B vaccination coverage, participants were asked “Have you ever had a vaccine for hepatitis?” and “What type(s) of hepatitis vaccine have you had?” Individuals could choose from “hepatitis A vaccine,” “hepatitis B vaccine,” “hepatitis A and B vaccine” or “I'm not sure which hepatitis vaccine I got.” Respondents who reported receiving “hepatitis B vaccine” or “hepatitis A and B vaccine” were classified as having received a vaccine for hepatitis B. Respondents who reported, “I'm not sure which hepatitis vaccine I got,” were excluded from the analysis. In this study, we evaluated the association of age, race/ethnicity, educational and insurance status, state of residence, lifetime history of sexual partners, anal intercourse, condom and drug use engaged in at last sex with the MRMSP, HIV testing behavior, having had a care visit with a healthcare provider in the last 12 months, and hepatitis B diagnosis and testing status with hepatitis B vaccination among MSM using chi-square tests of general association and bivariate odds ratios.

Men who reported having received a positive test result for hepatitis antibodies were excluded from bivariate analyses because hepatitis B vaccination is not recommended for individuals who have serological evidence of prior hepatitis B infection. Variables with bivariate chi-square p-values of less than 0.20 were entered into the initial multivariable model. The final main effects were determined using a backward selection algorithm wherein all variables not significant at a 5% alpha level were removed from the model. Once a main effects model containing all significant covariates was developed, the presence of two-way interactions was evaluated using a forward selection algorithm. We employed a Bonferroni-type correction in order to set the overall type I error rate for interactions at 5%. Multicollinearity of covariates and their interactions was assessed using the SAS macro COLLIN [19]. Collinearity was considered to be present if the condition index was >30.

We also compiled self-reported reasons why unvaccinated MSM were not vaccinated against hepatitis B. Men who reported never having been vaccinated against hepatitis B were asked to endorse all the reasons to explain why they had never been vaccinated from a specified list of reasons, as well as one “main” reason for lack of vaccination (for those participants who selected multiple reasons). Responses to this question were adapted from a study of viral hepatitis infection and vaccination among MSM that was conducted by Diamond et al on behalf of the US Young Men's Survey team [10]. If only one reason was reported, that reason was assumed to be the “main” reason for not being vaccinated against hepatitis B.

All analyses were performed using SAS 9.2 (SAS Institute, Cary, NC, USA).

## Results

**Figure 1:**
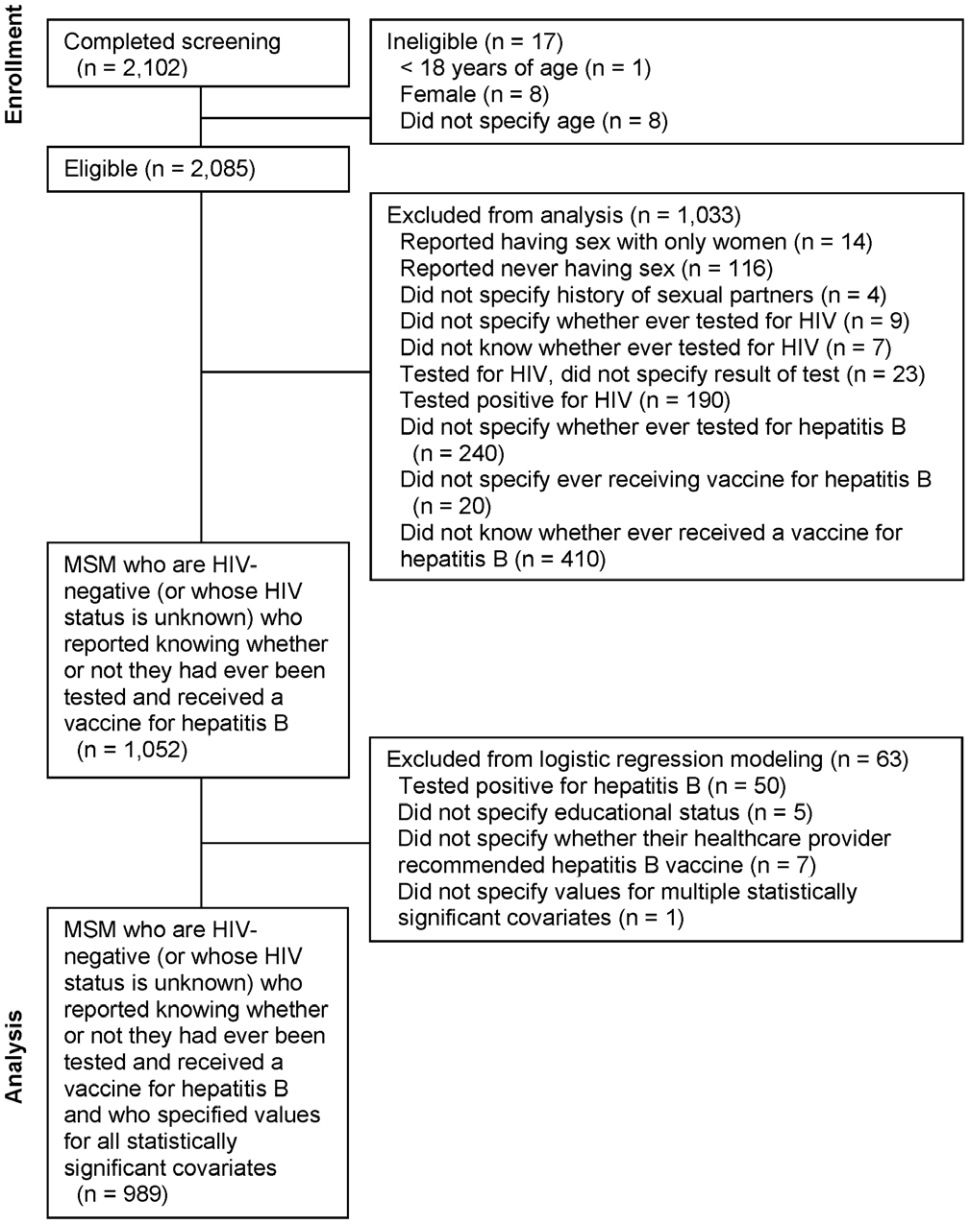
Derivation of study population from online cohort of MSM, United States, October–December 2010.

2,085 men aged 18 years of age or older were eligible to participate in our study. Of these, 134 participants (6.4%) who completed our questionnaire were excluded from analysis because they reported having sex with only women, reported never having sex, or did not specify a history of sexual partners. 39 individuals (1.9%) were also excluded because they did not report prior testing for HIV or because they were tested for HIV but chose not to report the HIV test result. In addition, 190 individuals (9.1%) reported testing positive for HIV. Finally, 670 individuals (32.1%) were excluded because they did not know or specify whether they had ever been tested for or vaccinated against hepatitis B. Thus, of 2,085 men 18 years of age or older who were eligible to complete our survey, 1,052 (50.5%) were included in our analyzed study population [Figure 1].

Table S1 describes the demographic and behavioral characteristics of the study population. Median age was 26. Based on examination of estimated logit plots, we divided age into three categories – 18–19, 20–31, and >31. More than 60% of the study population was non-Hispanic white, one fifth was non-Hispanic black, and one in every ten participants was Hispanic. Study participants were generally educated: approximately 80% reported at least having received an Associate's degree, attended technical school or completed some college coursework. More than half of all participants were insured by a private insurance company or a health maintenance organization. Unprotected anal intercourse at last sex with the most recent male sex partner (MRMSP) was common among study participants (46.7%); however, few participants reported drug use at last sex with their MRMSP (5.8%). More than 80% of all participants reported visiting a healthcare provider in the past year, but only one sixth of all participants reported that their healthcare provider recommended a hepatitis B vaccine during a recent visit.

Nearly three quarters of all participants reported ever being tested for hepatitis B; 50 (4.8%) reported they had ever been told by a healthcare provider that they were hepatitis B-positive. 679 (64.5%) reported ever being vaccinated against hepatitis B. Table S2 describes the bivariate association of each independent variable with vaccination status among the 1,002 hepatitis B-negative men in our study population. The odds of vaccination were approximately 50% lower for men aged greater than 31, compared to men aged 18–19 years. Higher educational attainment was associated with higher odds of vaccination: when compared to men with a high school education or less, the odds of vaccination were nearly twice as great for men who reported graduating from college, and were 60% higher for men who reported having received an Associate's degree, attended technical school or completed some college coursework.

Providers may screen their patients for hepatitis B antibodies in order to determine whether they are eligible to be vaccinated against the virus; hepatitis B testing was strongly associated with vaccination, as the odds of vaccination were 3.6 times higher among men who had ever been tested for hepatitis B, when compared to men who had never been tested. Although drug use at last sex was rare among all participants in our study, it remained associated with hepatitis B vaccination: the odds of vaccination were 50% lower among men who used drugs at last sex with their MRMSP, when compared to men who did not use drugs at last sex. Having been seen by a healthcare provider in the last year was also associated with hepatitis B vaccination, especially when the provider knew that the respondent had sex with men and when the provider recommended that the individual be vaccinated against hepatitis B. The odds of vaccination were 80% greater among men whose providers knew they had sex with men, when compared to men whose providers were unaware of their patient's male-male sexual behavior. The odds of vaccination were nearly five times higher among men whose providers recommended that they receive a vaccine for hepatitis B, when compared to individuals whose providers did not recommend vaccination.

Table S2 also presents the independent variables that remained associated with hepatitis B vaccination in the multivariable logistic regression model. Age, having been tested for hepatitis B, educational status, and having a provider recommendation for vaccination were all associated with significantly higher or lower odds of vaccination. (63 respondents who either reported being infected with hepatitis B [n = 50] or were missing information on statistically significant covariates [n = 13] were excluded from the model.) The odds of vaccination among MSM increased as men reported completing successively higher levels of education: receiving an Associate's degree, completing some college coursework, or having attended technical school was associated with a two-fold increase in the odds of vaccination, when compared to men with a high school education or less. By contrast, completing a college education was associated with a three-fold increase in the odds of vaccination. The odds of vaccination were four times greater among men whose healthcare providers recommended a vaccine for hepatitis B at a visit in the last year, when compared to men whose providers did not recommend vaccination. One significant interaction was identified: age interacted significantly with having been screened for antibodies to hepatitis B [Table S2]. Significant multicollinearity was not observed between any two independent predictors or their interactions.

Table 1 describes the reasons why HIV-negative men who reported they were not vaccinated against hepatitis B had never been vaccinated. More than half of these men reported that they had never been offered a vaccine by a healthcare provider, and one in five did not know that vaccination was recommended. Approximately one third of men were not aware that a hepatitis B vaccine was available and one quarter did not perceive themselves to be at increased risk of infection with hepatitis B. Overall, 90% of unvaccinated men reported one of the above reasons as the main reason why they had never been vaccinated against hepatitis B. Main reasons for not being vaccinated were generally similar to the reasons identified when men could endorse multiple reasons for never having been vaccinated [Table 1].

**Table 1:** Reasons for lack of hepatitis B vaccination among 323 unvaccinated, HIV- and hepatitis B-negative men who have sex with men, United States, October–December 2010.

	Any	Main
Reason not vaccinated	n (%)^a^	n (%)^b^,^c^
A vaccine was never offered to you	174 (53.9)	92 (41.8)
You didn't know there was a vaccine that prevents infection with hepatitis B	91 (28.2)	49 (22.3)
You believe yourself to be at low risk of contracting hepatitis B	80 (24.8)	43 (19.6)
You did not know you were eligible to receive a vaccine for hepatitis	68 (21.1)	13 (5.9)
A vaccine was offered to you, but you refused to receive it	12 (3.7)	8 (3.6)
There was no one assigned to you (i.e. doctor, nurse practitioner, etc) who could provide you with health care services, including immunizations	17 (5.3)	5 (2.3)
The vaccine for hepatitis B costs too much	13 (4.0)	5 (2.3)
You were unable to travel to a location that provides vaccinations because of circumstances that were out of your control (disability, live in rural area, etc)	4 (1.2)	2 (0.9)
You were incarcerated	2 (0.6)	2 (0.9)
You believed that you may already have symptoms of hepatitis B and therefore a vaccine would not work	3 (0.9)	1 (0.5)

a Percents may sum to more than 100 because respondents could specify more than 1 reason why they were not vaccinated against hepatitis B.

b Percents sum to more than 100 due to rounding.

c 103 respondents not vaccinated against hepatitis B either did not specify a reason for not being vaccinated, did not know why they were not vaccinated or, if they specified more than 1 reason for not being vaccinated, did not specify a main reason.

## Discussion

We found that nearly two thirds of HIV-negative participants who participated in a web-based sexual health survey of MSM between October and December 2010 reported ever being vaccinated against hepatitis B. Educational status, age, having been tested for hepatitis B, and provider-based recommendation of vaccination were significantly associated with self-reported receipt of vaccination. Based on these data, additional efforts are needed to improve hepatitis B vaccination rates in MSM. Understanding which factors are associated with self-reported vaccination will help clinicians and health promotion specialists tailor their immunization campaigns in a targeted effort to reach presently underserved segments of the population.

Previous studies of hepatitis B vaccination in MSM in the United States have estimated that the self-reported prevalence of vaccination is between 3% and 70% [8], [10], [12]–[14], [16]–[18], [20]. Although the results from our web-based survey approach the upper bound for the previously described, self-reported prevalence of vaccination in this population, it should be noted that a majority of other studies [8], [12], [13], [17], [18] found that no more than 40% of MSM had been vaccinated against hepatitis B. Furthermore, the high prevalence of vaccination reported by Siconolfi et al [14] in 2009 – 70% – may not be representative of all MSM in the United States because the authors solicited participation from individuals who were presumed to be more likely to be cognizant of their overall health and to seek to remain healthy.

Nevertheless, it is not implausible that we measured such a high prevalence of hepatitis B vaccination in our study population, given the demographics of our respondents. A hepatitis B vaccine was licensed in the United States in 1981 and universal infant vaccination recommendations have been in force since 1991 [4]. Therefore, men who are younger than 32 years of age, and especially those between the ages of 18 and 20, are more likely to have been vaccinated against hepatitis B. Men who are now between the ages of 18 and 20 would have likely been required to receive a hepatitis B vaccine in order to enter kindergarten, while men in their mid-twenties may have received a hepatitis B series as an early adolescent. Furthermore, men who pursued education beyond a high school or general equivalency diploma would have been likely to have received a vaccine for hepatitis B as a requirement for matriculation into a college or university. Unfortunately, however, one cannot assume that all men under the age of 32 received a hepatitis B vaccine as an infant, young child or adolescent. Like any other young person, a young MSM may not receive a vaccine for hepatitis B as a child because his parents may not be aware of vaccine recommendations or may object to vaccination because they have concerns over the safety of the vaccine.

Many prior studies have reported prevalences of vaccination in populations of MSM that were much older than the population surveyed in our study [8], [13], [14], [16], [20]. Thus, differences in vaccination prevalence between our study and prior reports may represent a cohort effect, because the predominantly older men in earlier studies were not children when the universal childhood vaccination recommendations were originally released in 1991 and subsequently revised in 1997. Compared to younger men, older men may more often only be vaccinated against hepatitis B because they are concerned that they may have been exposed to the virus and are seeking post-exposure prophylaxis.

Each of the factors significantly associated with hepatitis B vaccination among MSM in our study – age [8], [11], [12], [14], [20], educational status [11], [12], hepatitis B testing status [17] and history of provider recommendation of vaccination [8] – have also been found to be associated with vaccination in prior studies of MSM in the United States. In our study, we found that the odds of vaccination decreased with increasing age; however, men in their twenties and early thirties were more likely to be vaccinated if they had never been tested for hepatitis B, whereas older men were more likely to be vaccinated if they had ever been tested. A negative association between age and hepatitis B vaccination has been reported in many prior studies [8], [11], [12], [14], [20]. However, these studies have generally been conducted only in urban areas and have not included respondents from throughout the United States. Nationally representative surveys have enrolled only younger MSM (under the age of 30) [9]–[12]. However, most studies that were conducted more than ten years ago in select US cities, as well as over the Internet, examined self-reported hepatitis B vaccination prevalence in older MSM: most of their respondent profiles indicated a mean or median age of 30 years of age or older [13], [16], [20]. More recently, studies of vaccine prevalence among MSM have also been more likely to have older MSM respondents [8], [14]. We also found that men with higher levels of education were more likely to be vaccinated against hepatitis B. MacKellar et al reported a significant association between education and higher odds of vaccination in a model that also included age, city of residence, HIV testing status and diagnoses, the degree to which a man had ever told others that he was homosexual or bisexual, and whether he had a relationship with a healthcare provider. However, with respect to education, MacKellar et al modeled vaccination status as a function of a young man's current educational status [11]. As a result, it was not possible to assess the degree to which a man's level of education may simultaneously influence both his perception of infection risk associated with his sexual behavior and his likelihood of accepting vaccination. Also, the results of MacKellar et al were not directly comparable with ours. Weinbaum et al also reported a significant association between education and higher odds of vaccination in a model that included age, race, city of residence and whether a man had a regular source of healthcare [12]; however, they did not provide quantitative estimates of the association between education and receipt of vaccination.

We found that the odds of vaccination were more than four times higher among men whose healthcare providers recommended vaccination, when compared to men whose providers did not recommend vaccination. A 2009 study by Gilbert et al found that the odds of vaccination were nearly 18 times higher among men whose providers recommended hepatitis B vaccination, when compared to men whose providers did not recommend vaccination [8]; however, the bivariate association that they reported did not simultaneously control for the effects of confounding factors, as did our analysis.

Our study furthers knowledge of hepatitis B vaccination prevalence among HIV-negative MSM and factors significantly associated with vaccination because we evaluated self-reported vaccination among a group of MSM recruited from across the United States and from both urban and rural areas. Furthermore, the authors are aware of few studies of hepatitis B vaccination prevalence that examine this trend exclusively in HIV-negative individuals [15], [21]. In addition, we not only evaluated the relationship of covariates significantly associated with vaccination in a bivariate analysis, but we also developed a multivariable model to control for the effects of all statistically significant covariates simultaneously.

Among unvaccinated men in our study, we found that nearly nine in ten reported that their provider did not offer vaccination, they were unaware that a vaccine exists, or they did not perceive themselves to be at risk for hepatitis B infection, as the main reason they were never vaccinated against hepatitis B. Although previous studies have not reported the primary reason for lack of vaccination in their analyses and have instead described the range of reasons MSM report why they have never been vaccinated against the virus [8], [10]–[13], [17], the continued opportunity for providers to more actively promote disease prevention and vaccination in their consultations is clear. More than 40% of unvaccinated respondents in our study reported that the main reason that they were not vaccinated against hepatitis B was because a vaccine was never offered to them. Encouragingly, provider-recommended vaccination is effective at encouraging MSM to be vaccinated against the virus, as evidenced by a four-fold increase in the odds of vaccination among men in our study whose providers recommended vaccination, when compared with men whose providers did not.

Providers may not recommend vaccination to all eligible MSM because they are not aware that some patients engage in male-male sexual behavior. However, if a hepatitis B-susceptible MSM shares his male-male sexual behavior with his healthcare provider, the provider may be more likely to recommend vaccination [11]. Gilbert et al found that the odds of vaccination was more than two times higher among individuals who told their healthcare provider that they had sex with men, compared with individuals who did not [8]. In addition, a 2009 web-based study of provider-recommended HIV testing among MSM who saw a healthcare provider in the past 12 months found that men who shared their male-male sexual behavior with their providers were at least eight times more likely to be offered HIV testing, when compared to men who did not share their history of same-sex partners with their providers [22].

General delivery of health services to MSM, and especially prevention of HIV and acute hepatitis B infection, may therefore be improved if a healthcare provider knows that her patient engages in male-male sexual behavior. However, many MSM remain hesitant to disclose the fact that they have sex with other men to their providers [8], [11], [22]. Therefore, healthcare providers are encouraged to ask patients about whom they have sex with in a non-judgmental way, to include asking about male, female, or lack of any sexual partners. Providers should then screen all men who report male partners for serological evidence of past hepatitis B infection or vaccination, and offer vaccination to those men who are eligible to receive it. Providers and health promotion specialists should especially focus their efforts toward screening and vaccinating older, as well as socioeconomically disadvantaged, individuals.

There are several limitations inherent in our web-based sexual health survey of MSM in the United States. Because individuals were recruited over the Internet rather than in person by a trained interviewer, individuals who did not have access to the Internet during the study period were unable to participate and are not represented in the study population. In addition, a majority of our study participants were white. However, few prevalence studies of hepatitis B vaccination in MSM have chosen minority individuals as their population of interest [23], [24]. As more than half of our study population was under the age of 30, our results have limited generalizability to older individuals. Because our study was a cross-sectional survey of individual hepatitis B testing and vaccination status, demographic characteristics, sexual and non-sexual risk behaviors, and recent provider-participant interaction among HIV-negative MSM, we cannot determine whether factors significantly associated with vaccination preceded vaccination or whether other criteria for causality might have been met.

Because information on the outcome of interest and potentially associated covariates was ascertained by self-report, misclassification bias is likely to have occurred. Furthermore, although we restricted our study population to MSM who were HIV-negative or whose HIV status was unknown, there may have been some misclassification of self-reported HIV status. In addition, individuals may not remember whether they received the hepatitis B vaccine or because they are not aware that such a vaccine exists, they may mistake other shots or vaccines for the hepatitis B vaccine. We excluded participants who did not know or who chose not to specify whether they had ever received a hepatitis B vaccine. If these participants had otherwise been included in our study, we would have been required to assume that they were unvaccinated; as a result, the proportion of vaccinated individuals would have been incorrectly underestimated. However, it is not possible to estimate whether potential recall bias among individuals included in our study population – who reported knowing whether or not they were vaccinated against hepatitis B – led to an underestimation or overestimation of the prevalence of vaccination in this population.

In our study, we measured the prevalence of sexual and non-sexual risk behaviors that occurred at last sex with a study participant's MRMSP; however, as an example, a man's use of drugs at last sex with his MRMSP is not necessarily indicative of his typical drug use behavior during sex. Engaging in these high-risk sexual and non-sexual risk behaviors over long periods of time may be more significantly associated with vaccination than was engaging in these behaviors at last sex. Future studies could address this limitation by developing sexual and non-sexual risk behavior questions that incorporate an ordinal scale and evaluate whether MSM have engaged in such behaviors for more prolonged periods of time.

In addition, many of the reportable sexual and non-sexual risk behaviors, such as unprotected anal intercourse and drug use, are not often perceived to be socially desirable, and individuals may be less likely to report engaging in these activities. The prevalences of these behaviors in the population are likely to be underestimated. However, we do not have any reason to believe that men who had been vaccinated against hepatitis B were any more or less likely than men who had never been vaccinated to underreport socially undesirable behaviors. Therefore, it remains unlikely that either unprotected anal intercourse or drug use during last sex (or both) with the MRMSP actually have a significant association with receipt of hepatitis B vaccination that was obscured by this underreporting.

In addition to the limitations above, we recognize that internet-based surveys likely result in biased samples of men with respect to socioeconomic status, and often under-represent racial/minority MSM. Less gay-identified men would likely not have been reached by our advertising, which targeted Facebook participants based on their self-identified interest in men. Furthermore, because we did not have face to face contact with our participants, we cannot rule out the possibility that some of our respondents were not as they represented themselves. For example, women or heterosexual men could have responded to our survey, presenting themselves as MSM. However, we recruited respondents by targeting them for advertising based on their Facebook profile. Participants could not access the survey by simply entering a public URL. Therefore, we think it unlikely that women or heterosexual men created Facebook profiles identifying themselves as gay men, simply to gain access to an unincentivized survey. Ideally, one might seek to validate self-reported information on previous hepatitis testing or vaccination in medical records to reduce the possibility of misclassification of our outcomes.

Nearly one-third of HIV-negative MSM who participated in a web-based survey of their sexual and non-sexual life experiences reported never having been vaccinated against hepatitis B. Approximately 90% of these men reported not being vaccinated because they did not believe themselves to be at high risk of being infected with hepatitis B, or because they did not know about the availability of a vaccine for or were never offered vaccination against hepatitis B. Because men whose providers recommended vaccination against hepatitis B were four times more likely to be vaccinated against the virus than men whose providers did not recommend vaccination, providers should view consultations with their male patients who have sex with men as meaningful opportunities to offer vaccination to vulnerable patients. In addition, according to our data, providers and health promotion specialists should target their vaccination campaigns toward older MSM and those MSM of lower socioeconomic status.

## Supporting Information

Table S1 **Demographic and behavioral characteristics of 1,052 HIV-negative men who have sex with men who reported knowing whether or not they had ever been tested or received a vaccine for hepatitis B, United States, October–December 2010.**
(DOC)

Table S2 **Demographic and behavioral characteristics of 1,002 HIV- and hepatitis B-negative men who have sex with men who reported knowing whether or not they had ever received a vaccine for hepatitis B, United States, October–December 2010.**
(DOC)
