# Supplementary material for: Factors Associated with Self-Reported HBV Vaccination among HIV-Negative MSM Participating in an Online Sexual Health Survey: A Cross-Sectional Study
Source: PLoS One. 2012 Feb 17;7(2):e30609. doi: 10.1371/journal.pone.0030609 (PMC3281854; doi:10.1371/journal.pone.0030609)
Supplement: Table S1 — Demographic and behavioral characteristics of 1,052 HIV-negative men who have sex with men who reported knowing whether or not they had ever been tested or received a vaccine for hepatitis B, United States, October–December 2010. (DOC) [file pone.0030609.s001.doc]

Table S1. Demographic and behavioral characteristics of 1,052 HIV-negative men who have sex with men who reported knowing whether or not they had ever been tested or received a vaccine for hepatitis B, United States, October – December 2010.

| Characteristic | Value | n (%) |
| --- | --- | --- |
| Age (years)^a^ | >31 | 397 (37.7) |
|  | 20-31 | 506 (48.1) |
|  | 18-19 | 149 (14.2) |
| Race/ethnicity^a,b,c^ | Hispanic | 100 (9.8) |
|  | Non-Hispanic Black/African American | 208 (20.3) |
|  | Non-Hispanic Other^d^ | 88 (8.6) |
|  | Non-Hispanic White | 629 (61.4) |
| Educational status^a,c,e^ | College, post graduate or professional school | 372 (35.6) |
|  | Some college, Associate’s degree, and/or technical school | 467 (44.7) |
|  | High school or less | 207 (19.8) |
| Insurance status^a,c,f^ | Private/HMO | 558 (55.6) |
|  | Public | 80 (8.0) |
|  | Other^g^ | 73 (7.3) |
|  | None | 293 (29.2) |
| US Census region^a,c,h^ | Midwest | 217 (20.8) |
|  | Northeast | 171 (16.4) |
|  | West | 243 (23.3) |
|  | South | 413 (39.6) |
| Ever tested for hepatitis B^a^ | Yes | 787 (74.8) |
| Hepatitis B status^a^ | Told positive for hepatitis B | 50 (4.8) |
|  | Vaccinated | 679 (64.5) |
|  | Not vaccinated | 323 (30.7) |
| Ever tested for HIV^a^ | Yes | 905 (86.0) |
| Anal intercourse at last sex^a,c,i^ | Yes, unprotected by condoms | 486 (46.7) |
|  | Yes, protected by condoms | 262 (25.2) |
|  | No | 293 (28.2) |
| Drug use at last sex^a^,^j^ | Yes | 60 (5.8) |
| Ever had sex with a woman^a^ | Yes | 456 (43.4) |
| Visited a healthcare provider in the last 12 months^a,k^ | Yes | 888 (84.7) |
| Healthcare provider aware of patient’s status as a man who has sex with men at visit in last 12 months^l^ | Yes | 490 (47.0) |
| Healthcare provider recommended a vaccine for hepatitis B at visit in last 12 months^a,m^ | Yes | 173 (16.6) |

^a^Chi-square p-value ≤ 0.05

^b^27 respondents did not specify race/ethnicity

^c^Percents sum to more than 100 due to rounding

^d^Includes Asian/Pacific Islander, American Indian/Alaska Native, multi-racial and other self-reported racial groups

^e^6 respondents did not specify educational status

^f^48 respondents did not specify insurance status

^g^Includes Tricare/Champus, Veteran’s Administration coverage, and other self-reported types of insurance coverage

^h^8 respondents did not specify state of residence

^i^11 respondents did not specify having anal sex at last sex or frequency of condom use at last sex

^j^14 respondents did not specify whether they used drugs at last sex

^k^4 respondents did not specify whether they visited a healthcare provider in the last 12 months

^l^9 respondents did not specify whether their healthcare provider knows they have sex with men

^m^8 respondents did not specify whether their healthcare provider recommended that they receive a vaccine for hepatitis B
