# Supplementary material for: Factors Associated with Self-Reported HBV Vaccination among HIV-Negative MSM Participating in an Online Sexual Health Survey: A Cross-Sectional Study
Source: PLoS One. 2012 Feb 17;7(2):e30609. doi: 10.1371/journal.pone.0030609 (PMC3281854; doi:10.1371/journal.pone.0030609)
Supplement: Table S2 — Demographic and behavioral characteristics of 1,002 HIV- and hepatitis B-negative men who have sex with men who reported knowing whether or not they had ever received a vaccine for hepatitis B, United States, October–December 2010. (DOC) [file pone.0030609.s002.doc]

Table S1. Demographic and behavioral characteristics of 1,002 HIV- and hepatitis B-negative men who have sex with men who reported knowing whether or not they had ever received a vaccine for hepatitis B, United States, October – December 2010.

|  |  | Ever received hepatitis B vaccination | | Crude odds ratio | Adjusted odds ratio |
| --- | --- | --- | --- | --- | --- |
| Characteristic | Value | n (%) | | (95% CI) | (95% CI)^a^ |
|  |  | Yes | No |  |  |
| Age (years) | >31 | 205 (57.6) | 151 (42.4) | 0.5 (0.3, 0.8) | Ever tested for hepatitis B: 0.3 (0.1, 0.5)^b^ |
|  |  |  |  |  | Never tested for hepatitis B: 0.1 (0, 0.3)^b^ |
|  | 20-31 | 365 (73.4) | 132 (26.6) | 1.0 (0.7, 1.5) | Ever tested for hepatitis B: 0.5 (0.3, 1.0)^b,c^ |
|  |  |  |  |  | Never tested for hepatitis B: 0.9 (0.5, 1.6)^b^ |
|  | 18-19^d^ | 109 (73.2) | 40 (26.9) | Referent | Referent |
| Race/ethnicity^e^ | Hispanic | 67 (69.8) | 29 (30.2) | 1.0 (0.6, 1.7) | – |
|  | Non-Hispanic Black/African American^d^ | 121 (62.1) | 74 (38.0) | 0.7 (0.5, 1.0)^f^ | – |
|  | Non-Hispanic Other^g^ | 61 (72.6) | 23 (27.4) | 1.2 (0.7, 2.0) | – |
|  | Non-Hispanic White^d^ | 415 (69.1) | 186 (31.0) | Referent | Referent |
| Educational status^h^ | College, post graduate or professional school | 255 (72.0) | 99 (28.0) | 1.9 (1.3, 2.7) | 2.9 (1.9, 4.4) |
|  | Some college, Associate’s degree, and/or technical school | 305 (68.7) | 139 (31.3) | 1.6 (1.1, 2.2) | 2.1 (1.4, 3.1) |
|  | High school or less | 115 (58.1) | 83 (41.9) | Referent | Referent |
| Insurance status^i^ | Private/HMO | 369 (69.5) | 162 (30.5) | 1.2 (0.9, 1.6) | – |
|  | Public | 45 (59.2) | 31 (40.8) | 0.7 (0.4, 1.2) | – |
|  | Other^j^ | 42 (64.6) | 23 (35.4) | 0.9 (0.5, 1.6) | – |
|  | None | 187 (66.3) | 95 (33.7) | Referent | Referent |
| US Census region^k^ | Midwest | 140 (68.3) | 65 (31.7) | 1.2 (0.8, 1.7) | – |
|  | Northeast | 114 (69.9) | 49 (30.1) | 1.3 (0.9, 1.9) | – |
|  | West | 165 (71.4) | 66 (28.6) | 1.4 (1.0, 2.0)^l^ | – |
|  | South | 256 (64.5) | 141 (35.5) | Referent | Referent |
| Ever tested for hepatitis B | Yes | 557 (75.6) | 180 (24.4) | 3.6 (2.7, 4.9) | – |
|  | No | 122 (46.0) | 143 (54.0) | Referent | Referent |
| Ever tested for HIV | Yes | 591 (68.9) | 267 (31.1) | 1.4 (1.0, 2.0)^m^ | – |
|  | No | 88 (61.1) | 56 (38.9) | Referent | Referent |
| Anal intercourse at last sex^n^ | Yes, unprotected by condoms | 321 (69.0) | 144 (31.0) | 1.3 (1.0, 1.8)^o^ | – |
|  | Yes, protected by condoms | 176 (69.8) | 76 (30.2) | 1.4 (0.9, 1.9) | – |
|  | No | 173 (63.1) | 101 (36.9) | Referent | Referent |
| Drug use at last sex^p^ | Yes^d^ | 31 (53.5) | 27 (46.6) | 0.5 (0.3, 0.9) | – |
|  | No | 638 (68.6) | 292 (31.4) | Referent | Referent |
| Ever had sex with a woman | Yes | 277 (65.2) | 148 (34.8) | 0.8 (0.6, 1.1) | – |
|  | No | 402 (69.7) | 175 (30.3) | Referent | Referent |
| Visited a healthcare provider in the last 12 months^q^ | Yes | 581 (68.9) | 262 (31.1) | 1.4 (1.0, 2.0)^r^ | – |
|  | No^d^ | 94 (60.7) | 61 (39.4) | Referent | Referent |
| Healthcare provider aware of patient’s status as a man who has sex with men at visit in last 12 months^s^ | Yes | 344 (74.5) | 118 (25.5) | 1.8 (1.4, 2.4) | – |
|  | No | 329 (61.8) | 203 (38.2) | Referent | Referent |
| Healthcare provider recommended a vaccine for hepatitis B at visit in last 12 months^t^ | Yes | 141 (89.2) | 17 (10.8) | 4.8 (2.8, 8.0) | 4.2 (2.4, 7.4) |
|  | No | 531 (63.5) | 305 (36.5) | Referent | Referent |

^a^Multivariate logistic regression model does not contain 13 observations that were missing information on statistically significant covariates

^b^Adjusted odds ratios account for the presence of statistical interaction between age and hepatitis B testing status

^c^Upper bound of confidence interval is 1.04 (p = 0.07) and is rounded down to 1.0

^d^Percents sum to more than 100 due to rounding

^e^26 respondents did not specify race/ethnicity

^f^Upper bound of confidence interval is 1.03 (p = 0.07) and is rounded down to 1.0

^g^Includes Asian/Pacific Islander, American Indian/Alaska Native, multi-racial and other self-reported racial groups

^h^6 respondents did not specify educational status

^i^48 respondents did not specify insurance status

^j^Includes Tricare/Champus, Veteran’s Administration coverage, and other self-reported types of insurance coverage

^k^6 respondents did not specify state of residence

^l^Lower bound of confidence interval is 0.97 (p = 0.07) and is rounded up to 1.0

^m^Lower bound of confidence interval is 0.98 (p = 0.07) and is rounded up to 1.0

^n^11 respondents did not specify having anal sex at last sex or frequency of condom use at last sex

^o^Lower bound of confidence interval is 0.95 (p = 0.10) and is rounded up to 1.0

^p^14 respondents did not specify whether they used drugs at last sex

^q^4 respondents did not specify whether they visited a healthcare provider in the last 12 months

^r^Lower bound of confidence interval is 1.01 (p = 0.04) and is rounded down to 1.0

^s^8 respondents did not specify whether their healthcare provider knows they have sex with men

^t^8 respondents did not specify whether their healthcare provider recommended that they receive a vaccine for hepatitis B
